# Association of steroid regimens and withdrawal with relapse risk in myasthenia gravis: a real-world cohort study

**DOI:** 10.3389/fneur.2026.1792705

**Published:** 2026-05-13

**Authors:** Yangyu Huang, Ying Tan, Jingwen Yan, Yuzhou Guan

**Affiliations:** Department of Neurology, Peking Union Medical College Hospital, Peking Union Medical College, Chinese Academy of Medical Sciences, Beijing, China

**Keywords:** cohort study, corticosteroids, drug tapering, myasthenia gravis, relapse

## Abstract

**Background:**

Corticosteroids (steroids) are the first-line immunotherapy for myasthenia gravis (MG), but the optimal steroid dosing regimen and effects of discontinuation remain unclear. This study aimed to investigate the associations of steroid regimens and steroid withdrawal with relapse risk in patients on steroid monotherapy.

**Methods:**

This cohort study, based on a prospective registry, included MG patients who achieved minimal manifestations or better status for at least 6 months with steroid monotherapy. The primary outcome was relapse. Group-based trajectory modeling (GBTM) identified steroid regimens, and Cox proportional hazards models with propensity score matching (PSM) assessed the association of regimens and steroid withdrawal with relapse.

**Results:**

Among 209 patients followed for a median of 54.0 months, 113 (54.1%) experienced relapses. GBTM identified three regimens reflecting baseline disease severity: “High Start, Fast Taper” (Regimen 1), “Moderate Start, Gradual Taper” (Regimen 2), and “Low Start, Slow Taper” (Regimen 3). Compared to the high-risk Regimen 1, Regimen 2 (HR = 0.27, *p* < 0.001) and Regimen 3 (HR = 0.15, *p* < 0.001) were associated with significantly lower relapse risks. A statistically determined cut-off analysis found that requiring a maximum induction dose >0.83 mg/kg to achieve MM or better status was a predictor of relapse (HR = 1.54, *p* = 0.033). Sixty-eight (32.5%) withdrew steroids, and PSM showed that steroid withdrawal significantly increased relapse risk versus low-dose maintenance (HR = 1.64, *p* = 0.009).

**Conclusion:**

In this steroid-responsive population, patients requiring a maximum induction dose > 0.83 mg/kg to achieve MM or better status have a significantly higher risk of future relapse. Steroid withdrawal is associated with a higher relapse risk compared to low-dose maintenance and should be approached with caution.

## Introduction

Myasthenia gravis (MG) is an autoimmune disease caused by antibodies targeting antigen on motor endplate of the neuromuscular junction (1). Treatment strategies for MG include symptomatic management, immunosuppressive agents such as corticosteroids (steroids) and other immunosuppressants (2, 3), as well as molecular therapies like B cell-depleting agents, neonatal Fc receptor inhibitors and complement inhibitors (4). Thymectomy is effective in early-onset patients with positive acetylcholine receptor (AChR) antibodies or patients with thymoma (5–7). Despite diverse treatment options, oral steroids remain the first-line immunotherapy, achieving rapid improvement in up to 80% of patients (2, 8, 9).

However, there is no evidence-based consensus on optimal steroid dosing regimens. Initial dose may range from 1 mg/kg (prednisone equivalent) or gradually increased from 10 to 20 mg/day or 0.25 mg/kg until stable remission is achieved (2, 9–11). Subsequent steroid tapering aims to reduce the dose to the lowest effective level, though the rate of tapering varies across studies and centers (6, 7, 12–14). Although a randomized controlled trial (RCT) compared slow- and rapid-tapering regimens, its 15-month follow-up left long-term outcomes unclear (15). Real-world studies comparing different dosing regimens are limited by the individualized nature of steroid dosing in clinical practice, where patients may not strictly follow specific tapering protocols, complicating efforts to categorize them into discrete groups for comparison. Group-based trajectory modeling (GBTM), an extension of finite mixture modeling, addresses this issue by identifying clusters of patients with similar longitudinal patterns (16). In this study, we employed GBTM to identify distinct steroid regimens among patients who achieved clinical stability on steroid monotherapy, comparing their clinical characteristics and evaluating their associations with patient prognosis.

Research on whether immunotherapy can be safely discontinued in well-managed patients is limited. Guidelines suggest considering discontinuing immunotherapy after several years of stable remission (8). Su et al. (13) found that 35.3% of well-managed MG patients on steroid monotherapy experienced relapse, with a median relapse time of 4.0 months in a retrospective study. However, well-designed prospective studies comparing relapse risks and adverse events between steroid withdrawal and low-dose maintenance are still lacking.

In this study, we investigated the relationship between different steroid regimens and prognosis in a real-world cohort of steroid-responsive patients, and compared the relapse risks between steroid withdrawal and low-dose maintenance.

## Methods

### Study design and participants

This study was a single-center cohort study using prospective data from the Peking Union Medical College Hospital (PUMCH) MG registry, which enrolled all patients diagnosed with MG who had been followed for more than 6 months at PUMCH since January 2012. Data including demographics, clinical features of MG, comorbidities, treatment regimens and adverse events at baseline and follow-ups were prospectively collected in the registry.

For this study, we included all patients treated with oral glucocorticoids as monotherapy for maintenance therapy and achieved sustained minimal manifestation (MM) or better status following successful steroid tapering. The inclusion criteria were as follows: (1) age≥18 years; (2) diagnosis of MG according to the Chinese guideline for MG (17); (3) achieved MM or better status [MGFA Post-intervention Status (18)] with oral steroid monotherapy, without a history of other immunosuppressants or biologics (Intravenous methylprednisolone (IVMP) or intravenous immunoglobulin (IVIg) usage before initiating oral glucocorticoids was permitted); (4) successful tapering of oral steroids to below 0.25 mg/kg body weight (prednisone equivalent) without relapse for at least 6 months; and (5) a follow-up period of at least 12 months after tapering steroids to below 0.25 mg/kg. The establishment of the PUMCH MG registry and the research protocol for this study were both approved by the Ethics Committee of Clinical Research of Peking Union Medical College Hospital (Beijing, China). Written informed consent was obtained from all patients.

### Steroid dosing strategy

The steroid dosing strategy at our institution aligns with guideline recommendations and is designed to rapidly induce and subsequently maintain a status of minimal manifestation (MM) or better. An initial dose of 0.5–1.0 mg/kg (prednisone equivalent) is selected, with the precise dose being individualized according to clinical severity, comorbidities, patient tolerance, and shared decision-making. Patients undergo follow-up every 4 weeks during the initial treatment phase. Should a patient fail to achieve MM or better status within 4–8 weeks, the oral steroid dosage is progressively increased, not to exceed 1.0 mg/kg, until the therapeutic target is met.

Upon reaching the target status, the maximum dose is continued for a period of 4–8 weeks, after which tapering is initiated. The standard tapering protocol proceeds as follows:

1. When the daily dose is above 60 mg prednisone equivalent, a reduction of 5–10 mg is made every 4 weeks.
2. For daily doses between 40 and 60 mg, a 5 mg reduction is made every 4 weeks.
3. For daily doses between 20 and 40 mg, a 5 mg reduction is made every 4–8 weeks.
4. Once the daily dose is below 20 mg, it is further reduced by 2.5–5 mg every 3–6 months, according to the patient’s body weight and comorbidities. The goal is either to achieve a maintenance dose of 5–10 mg/day or to completely withdraw steroids within 18 to 36 months of treatment initiation.

The rate of tapering is individually tailored based on the patient’s clinical response and experienced adverse events.

### Data collection and outcome

Baseline data on demographics, clinical features at MG onset (including MGFA classification, MG-ADL score, and comorbidities), chest CT, and AChR and MuSK (muscle-specific kinase) antibody testing were collected. Patients with thymoma were recommended to undergo thymectomy. Steroid doses at each follow-up point, time to achieve MM or better, and adverse events [graded by Common Terminology Criteria for Adverse Events, CTCAE (19)] were recorded. Patients were instructed to monitor blood pressure, glucose, and weight at local hospitals. Overweight and obesity were defined as BMI ≥ 24 and ≥28, respectively (20). Bone mineral density was measured every 6–12 months using dual-energy X-ray absorptiometry (DXA, GE Lunar Prodigy, United States) to assess osteopenia and osteoporosis (21). Patients were followed every 1–3 months before achieving MM or better status and every 3–6 months after.

The primary outcome was the time to relapse after the initiation of oral steroids. Relapse was defined as meeting at least one of the following three criteria: (1) failure to maintain MM or better status; (2) an increase in Myasthenia gravis activities of daily living (MG-ADL) score to greater than 3 (22); (3) the need to resume cholinesterase inhibitors after achieving complete stable remission (CSR) or pharmacologic remission (PR) (18).

### Statistical analyses

Descriptive statistics for numerical variables are presented as mean ± SD or median (IQR), while categorical variables are shown as frequencies and percentages. Group comparisons were performed using appropriate tests, with the Holm-Bonferroni method for multiple comparison correction.

We used GBTM to identify distinct longitudinal patterns of steroid dosing regimens. The best-fitting model was selected based on Bayesian Information Criteria (BIC), average posterior probability (AvePP), and Odds of Correct Classification (OCC). Relapse risk was analyzed using Kaplan–Meier curves and a series of multivariate Cox proportional hazards (Cox PH) models. The proportional hazards assumption was tested using scaled Schoenfeld residuals, and multicollinearity was assessed with the Variance Inflation Factor (VIF). To compare outcomes between the steroid withdrawal and low-dose maintenance groups, we performed 1:1 propensity score matching (PSM) to balance key variables, including age at MG onset, gender, antibody status, thymectomy, thymoma, MG-ADL at steroid initiation, steroid regimen and time from steroid initiation to maintenance or withdrawal. Additionally, an Inverse Probability Treatment Weighting (IPTW) analysis was conducted on the full cohort as a sensitivity check. A two-sided *p*-value < 0.05 was considered statistically significant. All analyses were conducted using R software (version 4.3.2). A detailed description of the statistical analysis is available in the Supplementary Information. This study adhered to the Strengthening the Reporting of Observational Studies in Epidemiology (STROBE) reporting guidelines (23).

## Results

### Participant characteristics

**Figure 1:**
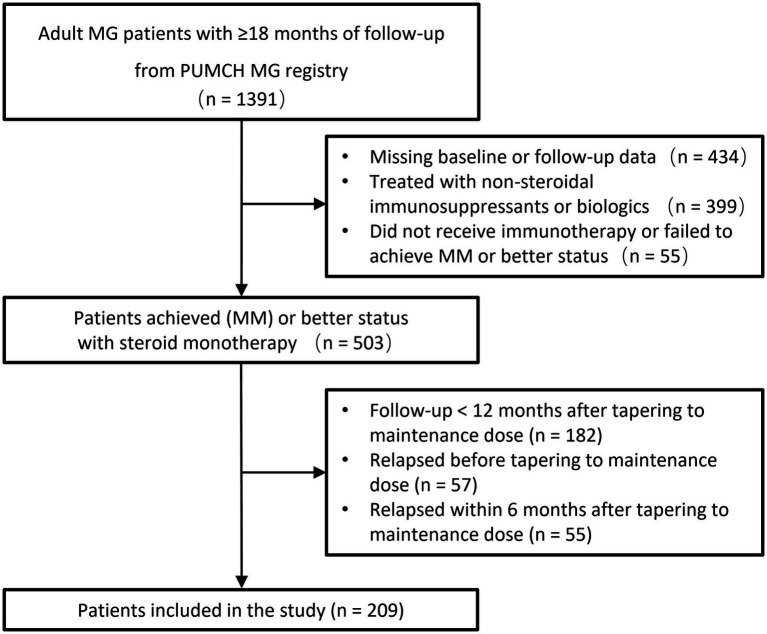
Flowchart of patient enrollment.

From the patients in the PUMCH MG registry followed between January 2012 and June 2024, 209 met inclusion criteria, as displayed in Figure 1. Baseline characteristics are presented in Table 1. Thymoma was diagnosed in 47 (22.5%) patients, and 56 (26.8%) underwent thymectomy. Twenty-seven (12.9%) had comorbid autoimmune diseases, mainly thyroid disorders (10 with Graves’ disease, 6 with Hashimoto’s thyroiditis).

**Table 1:** Baseline characteristics of all participants and groups of different steroid regimens.

Characteristics	All participants (*n* = 209)	Regimen 1: High start, fast taper (*n* = 30)	Regimen 2: Moderate start, gradual taper (*n* = 106)	Regimen 3: Low start, slow taper (*n* = 73)	*p*-value^a^
Female	110 (52.6%)	14 (46.7%)	57 (53.8%)	39 (53.4%)	0.778
Characteristics at MG Onset					
Age of MG onset	48.7 ± 14.6	45.2 ± 12.0	50.8 ± 14.7	47.0 ± 15.0	0.087
Early onset MG	96 (45.9%)	18 (60.0%)	42 (39.6%)	36 (49.3%)	0.109
MGFA classification					0.075
I	117 (56.0%)	13 (43.3%)	55 (51.9%)	49 (67.1%)	
II	51 (24.4%)	7 (23.3%)	27 (25.5%)	17 (23.3%)	
III	31 (14.8%)	6 (20.0%)	19 (17.9%)	6 (8.2%)	
IV	7 (3.3%)	3 (10.0%)	3 (2.8%)	1 (1.4%)	
V	3 (1.4%)	1 (3.3%)	2 (1.9%)	0	
MG-ADL	5.6 ± 3.5	6.5 ± 5.1	6.0 ± 3.5	4.6 ± 2.3	0.009
Other Autoimmune Diseases	27 (12.9%)	4 (13.3%)	13 (12.3%)	10 (13.7%)	0.966
Antibody					0.079
AChR	162 (77.5%)	22 (73.3%)	85 (80.2%)	55 (75.3%)	
MuSK	11 (5.3%)	4 (13.3%)	6 (5.7%)	1 (1.4%)	
Seronegative	36 (17.2%)	4 (13.3%)	15 (14.2%)	17 (23.3%)	
Thymoma	47 (22.5%)	11 (36.7%)	22 (20.8%)	14 (19.2%)	0.129
Thymectomy	56 (26.8%)	12 (40.0%)	24 (22.6%)	20 (27.4%)	0.164

^a^The *p*-values represent the significance of comparisons between the regimens.

AChR, acetylcholine receptor; MG-ADL, myasthenia gravis activities of daily living; MGFA, Myasthenia Gravis Foundation of America.

Treatment characteristics are presented in Table 2. The median time from MG onset to steroid initiation was 6.0 (3.0, 14.0) months; 31 patients (14.8%) received IVIg, and 9 (4.3%) received IVMP prior to steroids. The initial steroid dose was 0.57 (0.50, 0.84) mg/kg (prednisone equivalent). Fifty-four patients (25.8%) required dose escalation to reach MM or better status after failing to achieve MM or better status within 4–8 weeks of steroid initiation, while 65 (31.1%) eventually withdrew from steroids. The median follow-up was 54.0 (38.1, 73.1) months.

**Table 2:** Treatment characteristics across different steroid regimens.

Characteristics	All participants (*n* = 209)	Regimen 1: High start, fast taper (*n* = 30)	Regimen 2: Moderate start, gradual taper (*n* = 106)	Regimen 3: Low start, slow taper (*n* = 73)	*p*-value^a^
MG-ADL at steroid initiation	7.0 ± 3.9	9.0 ± 5.3	7.4 ± 3.7	5.5 ± 3.0	<0.001
Time from MG onset to steroid initiation (month)	6.0 (3.0, 14.0)	6.5 (4.3, 15.5)	6.0 (3.0, 14.8)	5.0 (3.0, 11.0)	0.339
Baseline weight (kg)	68.89 ± 12.24	65.03 ± 11.33	68.62 ± 12.35	70.87 ± 12.18	0.084
IVIg/IVMP before steroids initiation	40 (19.1%)	10 (33.3%)	24 (22.6%)	6 (8.2%)	0.006
Steroid dose (mg/kg)					
Initial dose	0.57 (0.50, 0.84)	0.87 (0.71, 1.00)	0.67 (0.54, 0.96)	0.50 (0.50, 0.56)	<0.001
Maximum dose	0.67 (0.54, 0.97)	1.00 (0.96, 1.02)	0.81 (0.64, 0.99)	0.51 (0.50, 0.56)	<0.001
Dose reduction from maximum					
6 months	0.32 ± 0.15	0.39 ± 0.19	0.36 ± 0.15	0.24 ± 0.08	<0.001
12 months	0.50 ± 0.20	0.65 ± 0.20	0.57 ± 0.18	0.33 ± 0.08	<0.001
18 months	0.57 ± 0.22	0.77 ± 0.18	0.65 ± 0.19	0.37 ± 0.08	<0.001
24 months	0.59 ± 0.22	0.81 ± 0.15	0.70 ± 0.19	0.39 ± 0.08	<0.001
Cumulative steroid dose by first taper (mg × month)	97.9 ± 88.6	187.9 ± 154.1	103.7 ± 68.8	52.5 ± 26.6	<0.001
Time from steroid initiation to first MM (month)	2.0 (1.0, 2.0)	2.0 (2.0, 3.0)	2.0 (1.0, 2.0)	1.0 (1.0, 2.0)	<0.001
Steroid withdrawal	68 (32.5%)	7 (23.3%)	36 (34.0%)	25 (34.2%)	0.508
Follow-up time (month)	54.0 (38.1, 73.1)	49.0 (31.2, 68.0)	57.5 (39.0, 75.1)	53.0 (42.0, 73.0)	0.455

Daily steroid doses were converted to equivalent dose of prednisone.

^a^The *p*-values represent the significance of comparisons between the regimens.

IVIg, intravenous immunoglobulin; IVMP, intravenous methylprednisolone; MG-ADL, Myasthenia Gravis Activities of Daily Living; MM, minimal manifestation.

### Identification of three steroids regimens using GBTM

**Figure 2:**
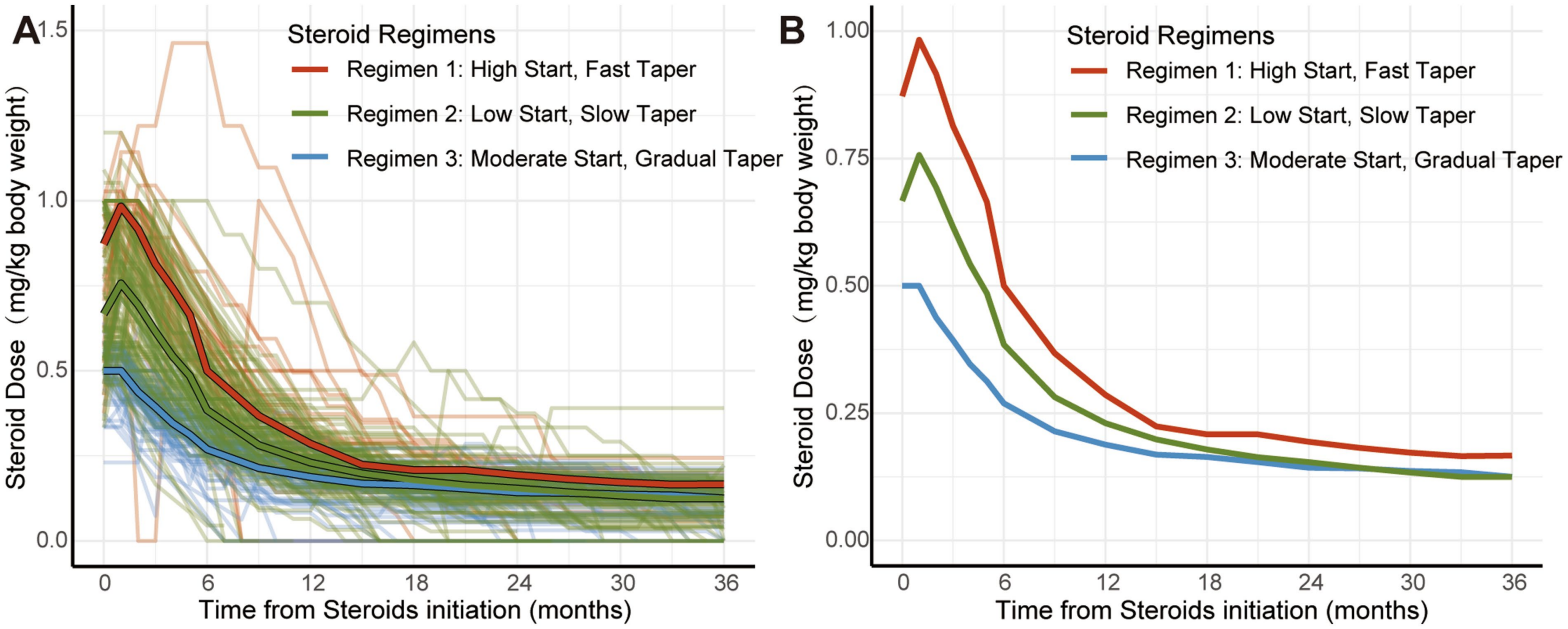
Trajectories of 3 steroid dosing regimens identified using group-based trajectory modeling. **(A)** Each faint-colored line represents the steroid dose per kg of body weight for individual patients over time, with colors indicating the regimen to which each patient belongs. **(B)** Median steroid dose per kg of body weight over time for each of the 3 regimens. Steroid doses were converted into equivalent prednisone doses.

In GBTM analysis, a three-group model provided the best fit to the data, with the lowest BIC and average posterior probabilities >0.90. The identified steroid regimen trajectory groups are shown in Figure 2, with baseline and treatment characteristics in Tables 1, 2. Regimen 1 (“High Start, Fast Taper”, *n* = 30, 14.4%) included patients with a median initial dose of 0.87 mg/kg and a maximum dose of 1.00 mg/kg prednisone equivalent. By 12 months, the dose was reduced by 0.65 ± 0.20 mg/kg from the maximum. 53.3% of patients required dose escalation after initiation, significantly higher than in Regimen 2 (31.1%) and Regimen 3 (6.8%) (adjusted *p* < 0.001 for both). Regimen 2 (“Moderate Start, Gradual Taper”, *n* = 106, 50.7%) had a median initial dose of 0.67 mg/kg and a maximum dose of 0.81 mg/kg, with a reduction of 0.57 ± 0.18 mg/kg at 12 months. Regimen 3 (“Low Start, Slow Taper”, *n* = 73, 34.9%) had a median initial dose of 0.50 mg/kg and a maximum dose of 0.51 mg/kg, with a reduction of 0.33 ± 0.08 mg/kg at 12 months. The time to taper below 0.25 mg/kg was 16.00 ± 7.8, 12.6 ± 4.8 and 8.8 ± 3.6 months for Regimens 1, 2, and 3, respectively (*p* < 0.001). There were no significant differences in the time to taper to 0.17 mg/kg (equivalent to 10 mg/day for a 60 kg patient) or 0.08 mg/kg (equivalent to 5 mg/day for a 60 kg patient) between the three groups (*p* = 0.133 and *p* = 0.314, respectively). The median time to taper to 0.17 mg/kg was 18.00 (13.00, 25.00) months, and to 0.08 mg/kg was 30.00 (19.75, 50.00) months.

The GBTM identifying different regimens aligns with our practice of tailoring steroid dosing based on disease severity and treatment response to achieve MM or better status quickly. As shown in Table 2 and Supplementary Figure 1, Regimen 1 had the highest disease severity, with significantly higher MG-ADL scores at steroid initiation compared to Regimens 2 and 3 (adjusted *p* < 0.001 for both). The median time from steroid initiation to first MM or better status for patients in Regimen 1 and Regimen 2 was 2.0 (2.0, 3.0) and 2.0 (1.0, 2.0) months, respectively, significantly longer than the 1.0 (1.0, 2.0) months for Regimen 3 (adjusted *p* < 0.01 for all comparisons). Additionally, the use of IVIg or IVMP prior to steroid initiation was significantly less frequent in Regimen 3 compared to Regimens 1 and 2 (adjusted *p* = 0.008 and 0.028, respectively). These findings suggest Regimen 1 was associated with more severe disease, requiring a more aggressive steroid approach, while Regimen 3 was associated with milder disease.

Adverse events observed during follow-up are shown in Supplementary Table 1. There were no significant differences in adverse events such as hypertension, diabetes, excess body weight, infection, and reduced bone density between the regimens. The overall infection rate was 20.1%, but the CTCAE grade of infection events in Regimen 1 was significantly higher than in the other two groups (*p* = 0.017), with grade 2 and 3 infections accounting for 85.7%. This suggests that high-dose steroids increase the risk of severe infections.

### Steroid regimens and other predictors as risk factors for relapse

During a median follow-up of 54.0 (38.1, 73.1) months, 113 (54.1%) patients experienced relapse. The cumulative probability of relapse after steroid initiation was 3.3% at 12 months, 11.6% at 24 months, 25.6% at 36 months and 40.5% at 48 months. The median time to relapse following steroid initiation was 56.0 (36.0, 81.0) months.

Among 117 patients with ocular MG at onset, 83 remained in the OMG stage at steroid initiation. During follow-up, 47 of these 83 patients (56.6%) relapsed, but only 9 (19.1%) progressed to generalized MG. Secondary generalization rates among relapsed patients did not significantly differ by regimen: 16.7% (1/6) in Regimen 1, 24.0% (6/25) in Regimen 2, and 12.5% (2/16) in Regimen 3 (*p* = 0.768). In the univariate Cox PH model, potential predictors of relapse (*p* < 0.10) included gender, the presence of other autoimmune diseases, baseline weight, cumulative steroid dose by the first taper, and the steroid regimen (Table 3). These variables, along with baseline and treatment characteristics that differed significantly across steroid regimens, and additional predictors previously reported or clinically considered relevant to MG prognosis (e.g., MG-ADL score, thymoma and thymectomy) were included in a comprehensive multivariate Cox PH model (13, 24–26). The multivariate model revealed that the steroid dosing regimen and comorbid autoimmune diseases remained independent predictors of relapse risk (Table 3). Compared to Regimen 1, both Regimen 2 (HR = 0.27, 95%CI 0.15–0.51, *p* < 0.001) and Regimen 3 (HR = 0.15, 95%CI 0.07–0.31, *p* < 0.001) were associated with a significantly lower risk of relapse. *Post hoc* pairwise comparisons further demonstrated that the relapse risk was significantly higher in Regimen 2 than in Regimen 3 (HR = 1.88, 95% CI 1.07–3.31, *p* = 0.025). Additionally, the presence of other autoimmune diseases was independently associated with a reduced risk of relapse (HR = 0.36, 95% CI 0.16–0.81, *p* = 0.013). Other covariates did not show independent associations with relapse in this fully adjusted model.

**Table 3:** Univariate and multivariate Cox proportional hazards model for predictors of relapse.

Predictors	Univariate model, HR (95% CI)	*p*-value	Multivariate model^c^, HR (95% CI)	*p*-value
Gender (male)	1.44 (1.11–1.88)	0.006	1.34 (0.99–1.80)	0.054
Age of MG onset	1.00 (0.99–1.01)	0.769	1.00 (0.99–1.01)	0.974
MGFA classification at MG onset^a^	0.91 (0.81–1.02)	0.116		0.364
MG-ADL score at MG onset	0.95 (0.89–1.01)	0.107		
Involved Muscle Groups at MG onset				
Ocular muscles	0.84 (0.57–1.26)	0.403		
Bulbar muscles	0.84 (0.64–1.11)	0.231	0.99 (0.63–1.58)	0.980
Limb muscles	0.94 (0.69–1.26)	0.667		
Respiratory muscles	0.59 (0.26–1.32)	0.198		
Other Autoimmune Diseases	0.44 (0.20–0.94)	0.033	0.36 (0.16–0.81)	**0.013**
Antibody (Seronegative as reference)				
AChR	0.88 (0.53–1.45)	0.614	1.00 (0.59–1.72)	0.988
MuSK	0.46 (0.15–1.35)	0.158	0.72 (0.23–2.29)	0.581
Thymoma	0.86 (0.54–1.35)	0.503	0.71 (0.30–1.68)	0.438
Thymectomy	0.97 (0.92–1.02)	0.182	1.15 (0.51–2.57)	0.743
Baseline weight	1.02 (1.01–1.03)	0.030	1.02 (0.98–1.05)	0.259
MG-ADL at steroids initiation	0.97 (0.92–1.02)	0.244	1.00 (0.93–1.09)	0.912
Time from MG onset to steroid initiation	1.00 (0.99–1.01)	0.640		
IVIg /IVMP before steroid initiation	0.76 (0.47–1.25)	0.283	0.84 (0.47–1.53)	0.561
Cumulative steroid dose by first taper^b^	0.83 (0.66–1.04)	0.097	0.66 (0.43–1.01)	0.057
Time from steroid initiation to first MM	0.89 (0.80–0.99)	0.043	0.89 (0.78–1.02)	0.085
Steroid regimen				
Regimen 1: High Start, Fast Taper	ref		ref	
Regimen 2: Moderate Start, Gradual Taper	0.55 (0.32–0.96)	0.035	0.27 (0.15–0.51)	**<0.001**
Regimen 3: Low Start, Slow Taper	0.48 (0.27–0.89)	0.018	0.15 (0.07–0.31)	**<0.001**

^a^MGFA classification was included in models as an ordinal categorical variable.

^b^Measured in units of per 100 mg·month.

^c^Multivariate model: Predictors with *p* < 0.10 in the univariate Cox PH model, along with baseline and treatment characteristics that differed significantly across steroid regimens, and additional predictors previously reported or clinically considered relevant to MG prognosis were included in the multivariate Cox PH model. Due to collinearity between the MG-ADL score at onset and at steroid initiation, only the MG-ADL score at steroid initiation was included.

AChR, acetylcholine receptor; IVIg, intravenous immunoglobulin; IVMP, intravenous methylprednisolone; MG-ADL, Myasthenia Gravis Activities of Daily Living; MGFA, Myasthenia Gravis Foundation of America; MuSK, muscle-specific kinase.

To assess whether thymoma influenced the association between steroid regimens and relapse risk, an additional Cox model was constructed with interaction terms (Steroid regimen × Thymoma), which revealed no significant interaction effects (*p* = 0.087 and 0.385), confirming that the impact of steroid regimens on prognosis remained consistent across patients with and without thymoma.

### Relapse risk increases when the daily steroid dose required to achieve MM or better status exceeds 0.83 mg/kg

As shown in Supplementary Figure 1 and Tables 1, 2, patients in Regimens 1 and 2 presented with higher MG-ADL scores at steroid initiation, required a higher maximum daily steroid dose to achieve MM or better status, and subsequently had a greater relapse risk compared to Regimen 3. Across all patients, the MG-ADL score at steroid initiation was significantly correlated with the maximum dose required to reach MM or better status (Spearman’s *r* = 0.37, *p* < 0.001), indicating that a higher peak dose was needed for more severe disease.

Given that the time required to taper to a low maintenance dose (0.17 mg/kg or 0.08 mg/kg) did not differ significantly between regimens, we hypothesized that the initial maximum dose required to achieve MM or better status was a key factor associated with long-term relapse. To identify a clinically relevant threshold, we performed a cut-off analysis using the surv_cutpoint function. The results revealed that patients requiring a maximum dose greater than 0.83 mg/kg to achieve MM or better status had a significantly increased risk of relapse (HR = 1.54, 95%CI 1.03–2.29, *p* = 0.033, see Supplementary Figure 2). To evaluate the structural stability of this data-driven threshold and minimize the risk of overfitting, we performed internal validation using 1,000 bootstrap resamples. The median optimal cut-off across the bootstrap distribution was 0.83 mg/kg (95% CI: 0.55–0.95), which aligns closely with our original estimate and confirms the robustness of this induction-peak threshold as a prognostic indicator.

### Effect of steroid usage duration on relapse in participants who withdrew steroids

Among all enrolled participants, 68 (32.5%) eventually withdrew steroids, and 48 of them (70.6%) experienced relapse. Apart from cumulative steroid dose, no significant baseline characteristic differences were observed between steroid maintenance and withdrawal groups (Supplementary Table 2). The Kaplan–Meier curve of relapse after steroid withdrawal is presented in Supplementary Figure 3. The median time to relapse after steroid withdrawal was 7.0 (3.0, 22.0) months, with a cumulative relapse rate of 35.8% at 6 months and 58.6% at 1 year. Univariate and multivariate Cox PH models were constructed to explore the impact of steroid duration on relapse after withdrawal (Supplementary Table 3). After adjusting for cumulative steroid dose and steroid regimen, no significant association was found between the duration of steroid use prior to withdrawal and the time to relapse post-withdrawal. This suggests that, regardless of prior steroid usage duration, patients who withdraw steroids face a high risk of relapse.

### Relapse risk between steroid maintenance and steroid withdrawal groups after PSM

**Figure 3:**
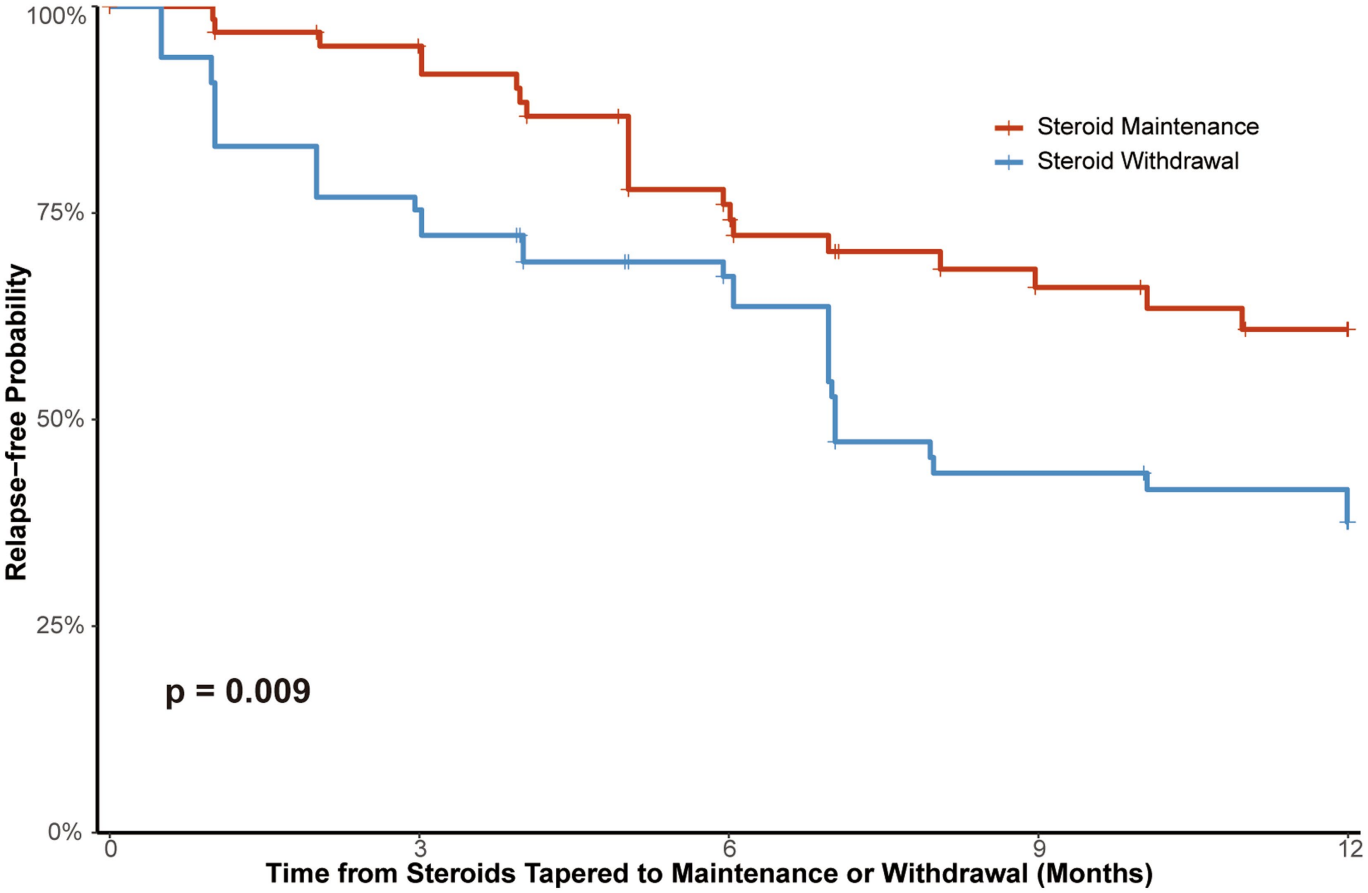
Kaplan–Meier curves of 1-year relapse probability in patients maintaining or withdrawing steroids after propensity score matching.

After PSM using variables including age at MG onset, gender, antibody status, thymectomy, thymoma, MG-ADL at steroid initiation, steroid regimen and time from steroid initiation to first taper to maintenance dose or withdrawal, 65 patients in the steroid maintenance group were matched with 65 patients in the steroid withdrawal group. The median maintenance dose in the steroid maintenance group was 5.00 (5.00, 10.00) mg. The characteristics of the two groups are shown in Supplementary Table 4. There were no significant differences between the two groups after matching, with standardized mean differences for all matching variables being <0.10. The Kaplan–Meier analysis revealed that during the 1-year follow-up period, the steroid withdrawal group had a significantly higher risk of relapse compared to the maintenance group (HR = 1.64, 95% CI 1.13–2.40, *p* = 0.009; Figure 3). The cumulative 1-year relapse rate was 62.4% in the withdrawal group, compared to 39.1% in the maintenance group. To further validate these findings, a sensitivity analysis using IPTW was performed on the entire cohort (*n* = 209). In the IPTW-adjusted cohort, all included covariates achieved excellent balance with SMDs < 0.10. The weighted Cox model consistently demonstrated that steroid withdrawal was an independent risk factor for 1-year relapse (HR = 1.78, 95% CI 1.29–2.46, *p* < 0.001). However, during this period, the steroid maintenance group had a higher risk of infection (*p* < 0.001), with 20 patients (30.8%) experiencing infection events, compared to 4 cases (6.2%) in the steroid withdrawal group. The adverse events during this period are presented in Supplementary Table 5.

## Discussion

This study evaluated the associations between different steroid regimens and steroid withdrawal and the risk of relapse in MG patients who achieved sustained MM or better status with steroid monotherapy, based on a real-world cohort with a median follow-up of 4.5 years. Using GBTM, we identified three distinct steroid regimens: “High Start, Fast Taper” (Regimen 1), “Moderate Start, Gradual Taper” (Regimen 2) and “Low Start, Slow Taper” (Regimen 3). These different regimens are consistent with our clinical practice of adjusting the early steroid dosing strategy based on disease severity and therapeutic response. Even after adjusting for confounders, Regimen 1 was associated with the highest relapse risk, followed by Regimens 2 and 3. This suggests that the steroid dosing regimen serves as a surrogate marker for underlying disease activity: patients requiring intensive induction to reach stability may harbor a more relapse-prone phenotype, whereas those achieving control with low-dose induction exhibit more stable long-term prognoses. Furthermore, the duration of steroid use before withdrawal did not influence the risk of relapse post-withdrawal. When comparing patients who withdrew from steroids with those on low-dose maintenance, the withdrawal group had a higher relapse rate, while the maintenance group faced a higher infection risk.

The GBTM identified distinct steroid regimens in a longitudinal real-world cohort, providing better insight into steroid use patterns than using average dosing. In our cohort, the initial steroid dose aligned with guideline recommendations based on disease severity, and dose escalation or tapering determined by the treatment response (2, 8, 9). Regimen 1, associated with the highest disease severity, had the largest initial dose and most frequent dose escalation, with a median maximum of 1.0 mg/kg. Conversely, Regimen 3, associated with the lowest severity, had a median maximum of 0.51 mg/kg. Compared to traditional tapering strategies [mean daily prednisone dose exceeding 30 mg/d at 15 months and 20 mg/d at 36 months (6, 27)], our regimen tapered more rapidly to a maintenance dose below 0.25 mg/kg in 11.8 ± 5.6 months, averaging 11.9 mg/d at 18 months.

Among patients who achieved MM or better status with steroid monotherapy, those on Regimen 1 (“High Start, Fast Taper”) had the highest relapse risk, while those on Regimen 3 (“Low Start, Slow Taper”) had the lowest, even after adjusting for confounders. Higher disease severity and rapid tapering are known risk factors for relapse (13, 26, 28). Patients in Regimen 1, with higher disease severity, required more frequent dose escalations after their initial dose, increasing both relapse risk and severe infections. In contrast, patients in Regimen 3, with milder disease, achieved sustained MM or better status through a slow taper and low-dose maintenance. Crucially, we identified a prognostic threshold of 0.83 mg/kg, defined as the maximum daily induction dose required to achieve MM or better status. Patients exceeding this threshold during the induction phase demonstrated a significantly higher risk of future relapse. This finding suggests that the “peak induction dose” may serve as a valuable predictor to identify high-risk individuals who might benefit from more prolonged maintenance or earlier introduction of non-steroidal immunosuppressants, rather than rapid withdrawal.

These results underscore the importance of individualized steroid regimens. For patients requiring higher therapeutic intensity (e.g., a maximum dose >0.83 mg/kg) to achieve MM or better status, the significantly increased risk of relapse and severe infection suggests that steroid monotherapy may be insufficient. In such cases, the early initiation of non-steroidal immunosuppressants is recommended as a “steroid-sparing” strategy to optimize symptom control and facilitate a safer tapering process, which aligns with multiple international management guidelines (8, 9, 29). However, our data also provide supportive evidence for low-dose steroid monotherapy as a viable maintenance approach for a subset of patients who achieve stability with lower initial doses. This is particularly relevant in clinical practice in China, where long-term steroid monotherapy remains highly prevalent (24). The choice of maintenance therapy is often dictated by socioeconomic factors; for instance, while azathioprine is widely available, its clinical compliance is frequently limited by slow onset and side effects. Conversely, immunosuppressants like tacrolimus and mycophenolate mofetil are often not covered by basic medical insurance for MG in many regions, and their high cost, which may exceed 50% of a household’s monthly disposable income in China (30), presents a significant barrier to long-term use.

Multivariate Cox PH models showed that comorbid autoimmune diseases were associated with a lower risk of relapse. In our cohort, 12.9% of patients had comorbid autoimmune diseases, primarily thyroid disorders. This frequency is consistent with the reported range of 13–22% in the MG population (24–26, 31) and is notably higher than the approximately 5% prevalence observed in the general population (32). The impact of autoimmune diseases on MG varies across studies, with some (particularly those focusing on generalized MG) identifying them as risk factors for relapse (24, 25, 33), while others suggest they may be associated with milder disease such as ocular MG and less exacerbation (34–36). Our cohort’s high proportion of ocular MG (56.0%) and longer follow-up may explain the results. Future research is needed to confirm the impact of comorbid autoimmune diseases on the long-term course of MG and to explore their differing roles across various MG subtypes.

We found that 70.6% of patients who withdrew steroids experienced relapse, with a significantly higher relapse rate compared to those on low-dose maintenance. Studies on relapse after discontinuing immunosuppressive therapy in MG are limited. Su et al. reported a 35.3% relapse rate among those who withdrew steroids, with a median relapse time of 4.0 months (13). Our study observed a higher relapse rate and a longer median time to relapse (7.0 months), possibly due to longer follow-up. Both our study and Su et al. found no association between the duration of steroid use prior to withdrawal and relapse (13), suggesting that even after years of MM or better status, the risk of short-term relapse remains high. This is consistent with previous findings that only 10% of patients achieve remission without immunosuppressive therapy (3, 37–39). Our results, along with these findings, suggest long-term maintenance therapy is reasonable in MG, and steroid withdrawal should be approached cautiously.

However, the use of low-dose steroids for long-term immunotherapy remains debated. Our findings indicate low-dose steroids can still increase infection risk. Corticosteroid-related side effects are influenced by both dosage and duration (3). Population-based studies suggest that daily prednisone doses below 5–7.5 mg can minimize most side effects, with Japanese guidelines targeting MM with a dose below 5 mg (29, 40, 41). Nonetheless, in our cohort, a median daily steroid dose of 5.0 mg significantly increased infection risk. This does not suggest that other immunosuppressants are safer for long-term use, as they also carry risks of infection, bone marrow suppression, or malignancies (3, 24). One study found that the side effect burden of immunosuppressants is similar to that of low-dose steroids using quantified tools to assess adverse events (42). This underscores the need for further research to determine the optimal maintenance therapy that balances efficacy and side effects.

Our study has limitations. First, it was a single-center study, and steroid regimens may differ across centers, though our regimen aligns with multiple guidelines. Additionally, our findings should be interpreted within the context of our inclusion criteria. This study specifically evaluated patients who were responsive to steroid monotherapy and achieved stable remission. Therefore, the generalizability to patients with refractory MG or those requiring other immunosuppressants or biologics remains to be established. Lastly, although we focused on treatment-related side effect and graded them using CTCAE, certain steroid-related side effects, such as depression and anxiety were not included in the registry. These side effects, which impact quality of life, are difficult to assess in routine practice (38, 39). Further multicenter studies are needed to explore the long-term prognostic impacts and side effects of different immunotherapies.

In conclusion, among steroid-responsive MG patients who achieve stability on monotherapy, our findings demonstrate that baseline disease activity and the steroid regimen are associated with long-term prognosis. Patients with higher initial disease severity often necessitate a higher maximum induction dose (approaching 1.0 mg/kg) to achieve a sustained MM or better status, which is linked to a significantly higher risk of future relapse. Specifically, requiring a maximum induction dose exceeding 0.83 mg/kg to reach MM or better status serves as a robust prognostic indicator for increased relapse risk. In contrast, patients who achieve sustained stability with a low-dose induction (e.g., 0.5 mg/kg) and a slow taper have a markedly lower risk of relapse. These findings underscore the importance of an individualized steroid treatment strategy. Furthermore, the duration of steroid use prior to withdrawal did not influence post-withdrawal relapse risk. Steroid withdrawal significantly increases the risk of relapse compared to maintaining a low-dose regimen. Therefore, discontinuation of immunotherapy, including steroids, should be approached with caution in MG patients who have achieved long-term stability.

## Data Availability

The datasets presented in this article are not readily available because of the restriction from the authors’ affiliation. Requests to access the datasets should be directed to Yuzhou Guan, guanyz001@163.com.
