# Supplementary material for: Association of steroid regimens and withdrawal with relapse risk in myasthenia gravis: a real-world cohort study
Source: Front Neurol. 2026 May 13;17:1792705. doi: 10.3389/fneur.2026.1792705 (PMC13212052; doi:10.3389/fneur.2026.1792705)
Supplement: Supplementary file 1 [file Data_Sheet_1.PDF]

Supplementary Information for

Association of Steroid Regimens and Withdrawal with Relapse Risk in Myasthenia Gravis: A Real-World Cohort Study

Yangyu Huang, M.D.[1], Ying Tan, M.D. [1], Jingwen Yan, M.D. [1] and Yuzhou Guan, M.D., Ph.D. [1*]

1 Department of Neurology, Peking Union Medical College Hospital, Peking Union Medical College, Chinese Academy of Medical Sciences, Beijing, China.

*** Corresponding author:**

Yuzhou Guan,

M.D., Ph.D., Department of Neurology, Peking Union Medical College Hospital, Peking Union Medical College, Chinese Academy of Medical Sciences, No. 1 Shuaifuyuan Wangfujing Dongcheng District, Beijing, China 100730

E-mail: guanyz001@163.com

Supplementary contents: Detailed statistical methods

Supplementary Figure 1 Comparison of Time to Achieve MM or Better Status and Disease Severity Across Different Steroid Regimens

Supplementary Figure 2 Impact of Maximum Steroid Dose Required to Achieve MM or Better Status on Relapse Risk

Supplementary Figure 3 Kaplan-Meier Curve of Relapse Following Steroid Withdrawal.

Supplementary Table 1 Side effects of different steroid regimens.

Supplementary Table 2 Characteristics of steroid maintenance and steroid withdrawal groups.

Supplementary Table 3 Effect of steroid usage duration on relapse in participants who withdrew steroids

Supplementary Table 4 Characteristics of steroid maintenance and steroid withdrawal groups after propensity score matching.

Supplementary Table 5 Side effects of steroids maintenance and steroids withdrawal groups after propensity score matching.

Detailed statistical methods

Descriptive statistics for numerical variables were presented as mean ± standard deviation (SD) for normally distributed data or median (interquartile range, IQR) for non-normally distributed data. Categorical variables were described using frequencies and percentages. For group comparisons, Student's t-test or one-way analysis of variance (ANOVA) was used for normally distributed continuous variables, while the Wilcoxon rank-sum test or Kruskal-Wallis test was applied for non-normally distributed continuous variables. The Holm-Bonferroni method was used to adjust for multiple comparisons.

To identify distinct longitudinal patterns of steroid dosing, we employed Group-Based Trajectory Modeling (GBTM). We estimated trajectory models with 2–4 trajectories based on the monthly steroid dose (converted to the equivalent prednisone dose per kg of body weight). For each group, a polynomial function of time (up to third order) was considered. Model selection was based on Bayesian Information Criteria (BIC), average posterior probability (AvePP), and Odds of Correct Classification (OCC). After determining the best-fitting model, we compared the clinical characteristics and steroid dosing patterns across different regimens.

We constructed Cox PH models to analyze the impact of different steroid regimens on the risk of relapse. Predictors of relapse were first identified through univariate Cox PH analysis. Variables with $p < 0.10$ in the univariate analysis, baseline characteristics showing significant inter-group differences, and additional predictors previously reported or clinically considered relevant to MG prognosis were included in the final fully adjusted multivariate Cox PH model. The proportional hazards assumption was verified via scaled Schoenfeld residuals, and multicollinearity was monitored using the Variance Inflation Factor (VIF), with a threshold of < 5.0 indicating no significant collinearity.

Subsequently, the surv_cutpoint function (R package 'survminer') was utilized to determine the optimal threshold for the maximum daily steroid dose required to achieve MM or better status based on maximally selected rank statistics. To assess the structural stability of the identified cut-off and minimize the risk of overfitting, we performed internal validation using 1,000 bootstrap resamples. The median optimal cut-off across the bootstrap distribution was calculated along with its 95% confidence interval.

To assess the impact of steroid withdrawal on the 1-year relapse rate, propensity score matching (PSM) between the steroid maintenance group and the steroid withdrawal group was performed using 1:1 matching within a caliper width of 0.2 standard deviations. The propensity score was calculated using a logistic regression model with prespecified variables including age at MG onset, gender, antibody status, thymectomy, thymoma, MG-ADL at steroid initiation, steroid regimen and time from steroid initiation to maintenance or withdrawal. Standardized mean differences (SMD) were calculated to assess the effectiveness of the matching, with values greater than 0.10 indicating significant imbalance. Furthermore, an Inverse Probability Treatment Weighting (IPTW) analysis was conducted as a sensitivity analysis to confirm the robustness of the results across the entire cohort (n=209). Weights were calculated based on the propensity scores and trimmed at the 99th percentile to stabilize the model and minimize the influence of extreme weights. The weighted average treatment effect (ATE) was then estimated using a weighted Cox PH model. A two-sided p value <0.05 was considered statistically significant. All analyses were performed using R software (version 4.3.2).

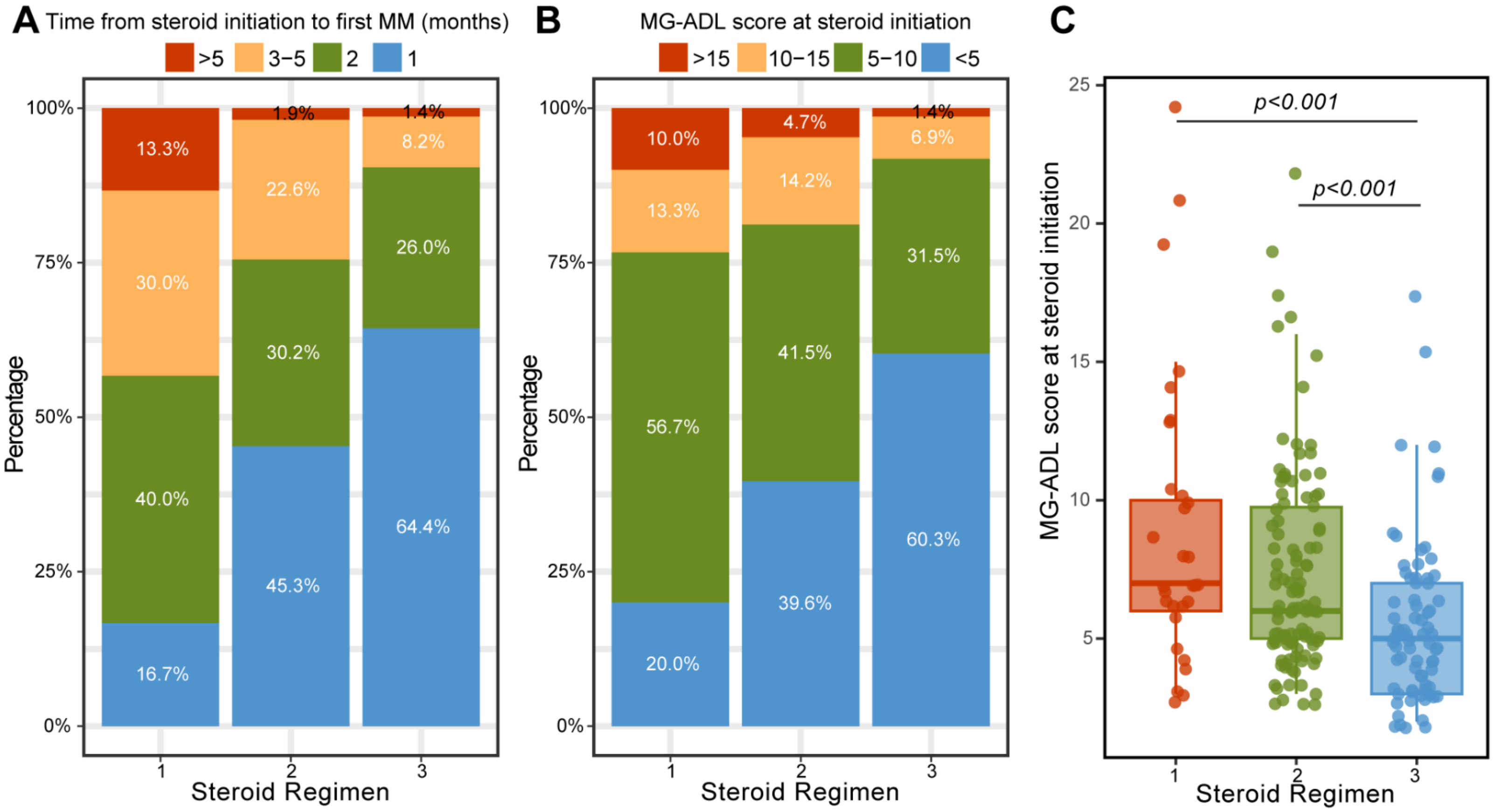

Supplementary Figure 1 Comparison of Time to Achieve MM or Better Status and Disease Severity Across Different Steroid Regimens

A: Distribution of the time from steroid initiation to first achieving Minimal Manifestation (MM) or better status for each steroid regimen.

B: Distribution of Myasthenia Gravis-Activities of Daily Living (MG-ADL) scores at steroid initiation for each steroid regimen.

C: Box plot comparing MG-ADL scores at steroid initiation across the three steroid regimens. The p-values represent significant differences between groups.

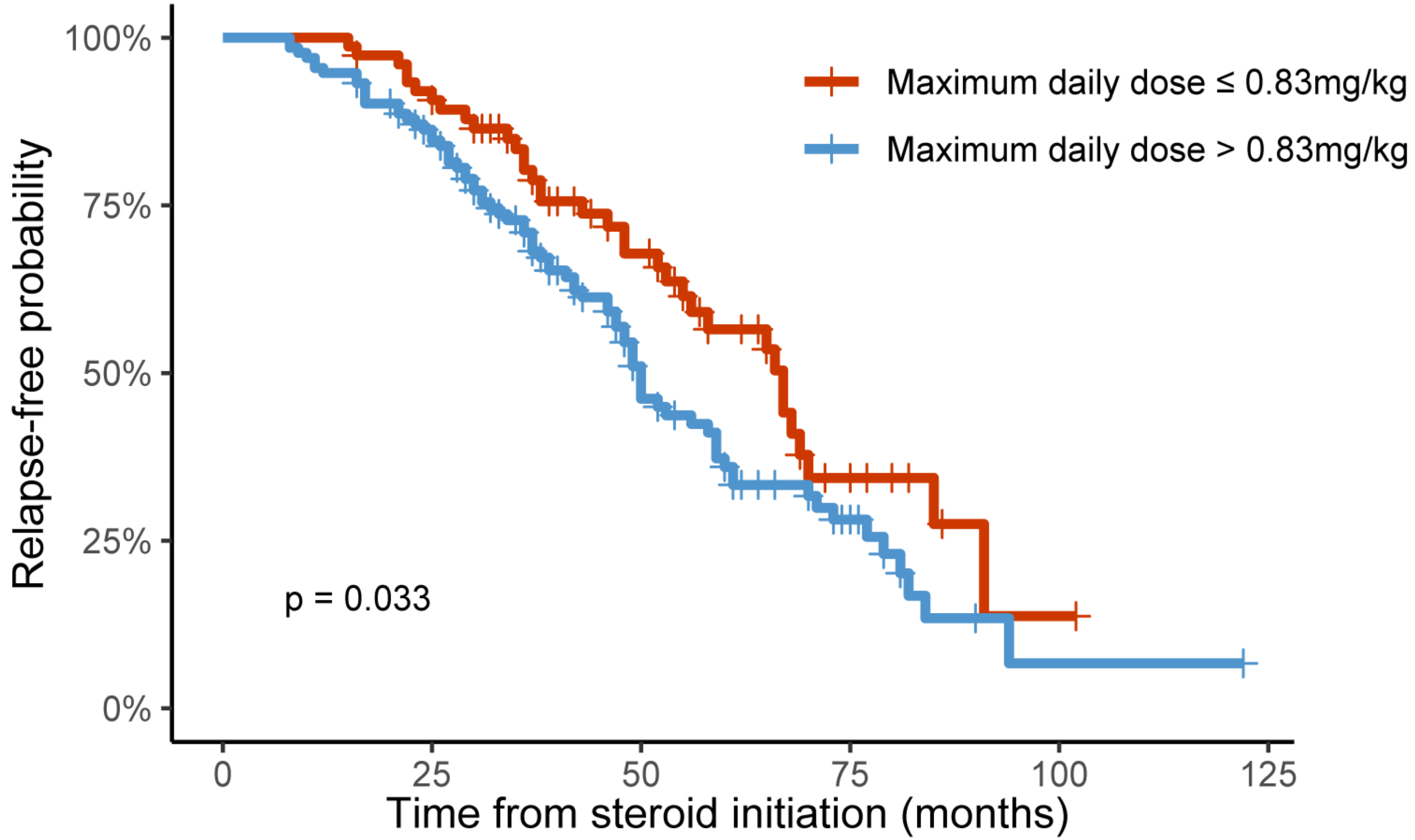

Supplementary Figure 2 Impact of Maximum Steroid Dose Required to Achieve MM or Better Status on Relapse Risk

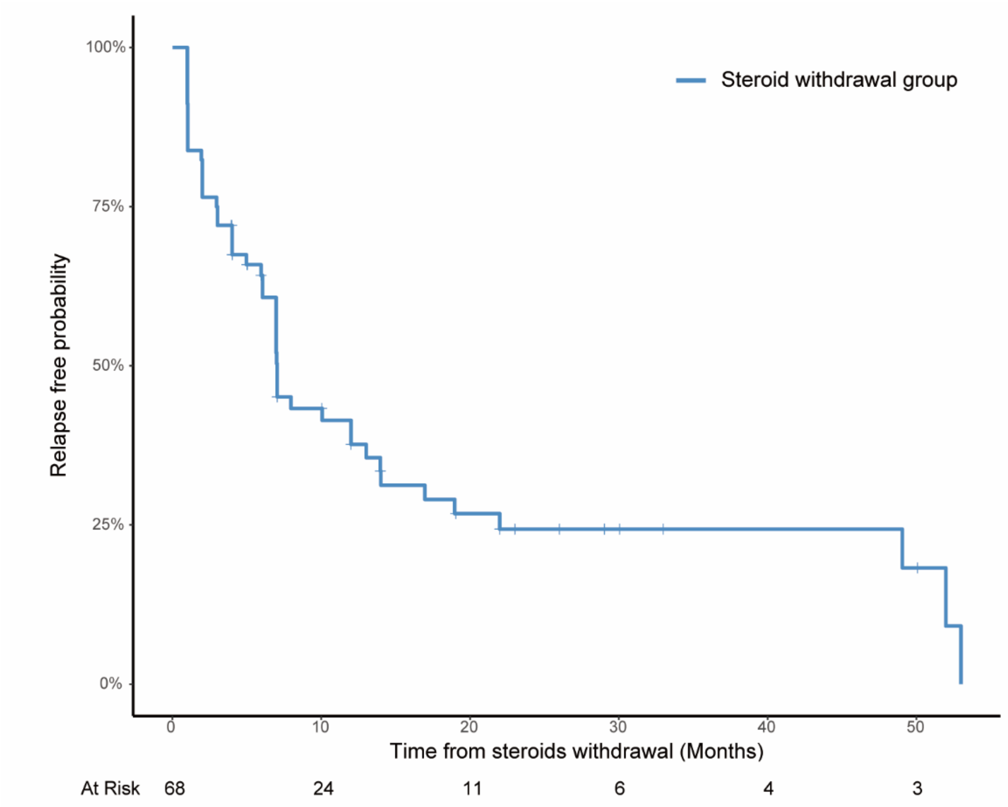

Supplementary Figure 3 Kaplan-Meier Curve of Relapse Following Steroid Withdrawal.

Supplementary Table 1 Side effects of different steroid regimens

	All participants (n = 209)	Regimen 1: High Start, Fast Taper (n = 30)	Regimen 2: Low Start, Slow Taper (n = 73)	Regimen 3: Moderate Start, Gradual Taper (n = 106)	p value
Hypertension	24 (11.5%)	4 (13.3%)	6 (8.2%)	14 (13.2%)	0.567
Diabetes	27 (12.9%)	2 (6.7%)	10 (13.7%)	15 (14.2%)	0.615
Insomnia requiring medication	30 (14.4%)	4 (13.3%)	10 (13.7%)	16 (15.1%)	0.960
Excess body weight					
Overweight	36 (17.2%)	4 (13.3%)	12 (16.4%)	20 (18.9%)	0.947
Obesity	26 (12.4%)	4 (13.3%)	10 (13.7%)	12 (11.3%)	
Infection	42 (20.1%)	8 (26.7%)	11 (15.1%)	23 (21.7%)	0.644
Specific infectious diseases					0.331
Pulmonary infection	7 (3.4%)	3 (10.0%)	2 (2.7%)	2 (1.9%)	
Urinary tract infection	6 (2.9%)	2 (6.7%)	3 (4.1%)	1 (0.9%)	
Skin infection	3 (1.4%)	1 (3.3%)	1 (1.4%)	1 (0.9%)	
Herpes zoster	3 (1.4%)	0 (0.0%)	1 (1.4%)	2 (1.9%)	
Gastrointestinal infection	3 (1.4%)	1 (3.3%)	1 (1.4%)	1 (0.9%)	
Tuberculosis	2 (1.0%)	0	1 (1.4%)	1 (0.9%)	
Central nervous system infection	1 (0.5%)	0	0	1 (0.9%)	
CTCAE of infection event					0.017

Grade 1	1 (0.5%)	1 (3.3%)	0	0	
Grade 2	18 (8.6%)	3 (10.0%)	9 (12.3%)	6 (5.7%)	
Grade 3	6 (2.9 %)	3 (10.0%)	0	3 (2.8%)	
Reduced bone density					
Osteopenia	54 (25.8%)	7 (23.3%)	17 (23.3%)	30 (28.3%)	0.644
Osteoporosis	42 (20.1%)	7 (23.3%)	12 (16.4%)	23 (21.7%)	
Osteoporotic Fractures	10 (4.8%)	1 (3.3%)	2 (2.7%)	7 (6.6%)	
Femoral head necrosis	5 (2.4%)	1 (3.3%)	0	4 (3.8%)	0.222
Cataract requiring surgery	10 (4.8%)	0	5 (6.9%)	5 (4.7%)	0.463

CTCAE, Common Terminology Criteria for Adverse Events.

Supplementary Table 2 Characteristics of steroid maintenance and steroid withdrawal groups

	All participants (n = 209)	Steroids maintenance (n = 141)	Steroids withdrawal (n = 68)	p value
Female	110 (52.6%)	72 (51.1%)	38 (55.9%)	0.613
Characteristics at MG Onset				
Age of MG onset	48.7 ± 14.6	48.6 ± 14.7	48.6 ± 14.5	0.993
Early onset MG	96 (45.9%)	66 (46.8%)	30 (44.1%)	0.828
MGFA classification				0.163
I	117 (56.0%)	71 (50.4%)	46 (67.7%)	
II	51 (24.4%)	38 (27.0%)	13 (19.1%)	
III	31 (14.8%)	24 (17.2%)	7 (10.3%)	
IV	7 (3.3%)	6 (4.3%)	1 (1.5%)	
V	3 (1.4%)	2 (1.4%)	1 (1.5%)	
MG-ADL score	5.6 ± 3.5	5.8 ± 3.6	5.3 ± 3.2	0.273
Other Autoimmune Diseases	27 (12.9%)	21 (14.9%)	6 (8.8%)	0.315
Antibody				0.636
AChR	162 (77.5%)	107 (75.9%)	55 (80.9%)	
MuSK	11 (5.3%)	9 (6.4%)	2 (2.9%)	
Seronegative	36 (17.2%)	25 (17.7%)	11 (16.2%)	
Thymoma	47 (22.5%)	32 (22.7%)	15 (22.1%)	1
Thymectomy	56 (26.8%)	38 (27.0%)	18 (26.5%)	1
Steroids regimen				0.278
Regimen 1: High Start, Fast Taper	30 (14.4%)	24 (17.0%)	6 (8.8%)	

Regimen 2: Low Start, Slow Taper	73 (34.9%)	47 (33.3%)	26 (38.2%)	
Regimen 3: Moderate Start, Gradual Taper	106 (50.7%)	70 (49.7%)	36 (52.9%)	
Cumulative steroids dose (mg × month)	662.1 ± 284.5	708.2 ± 279.6	566.6 ± 272.2	<0.001

Supplementary Table 3 Effect of steroid usage duration on relapse in participants who withdrew steroids

Duration of steroid usage before withdrawal	Model 1<br>HR (95% CI)	p value	Model 2[‡]<br>HR (95% CI)	p value	Model 3[§]<br>HR (95% CI)	p value
Whole group[†]	1.00 (0.98-1.01)	0.529	0.99 (0.96-1.01)	0.256	0.99 (0.98-1.01)	0.354
<12 months	ref		ref		ref	
12-24 months	0.98 (0.37-2.53)	0.972	0.90 (0.32-2.54)	0.835	0.74 (0.26-2.07)	0.567
24-36 months	0.93 (0.35-2.44)	0.882	0.84 (0.29-2.43)	0.749	0.83 (0.31-2.22)	0.712
36-48 months	1.43 (0.53-3.85)	0.484	1.20 (0.34-4.22)	0.778	1.18 (0.41-3.43)	0.757
>48 months	0.83 (0.34-2.03)	0.689	0.66 (0.17-2.61)	0.553	0.67 (0.23-1.98)	0.427

† Using withdrawal duration as a continuous covariate

‡ Model 2: Adjusted for cumulative dose.

§ Model 3: Adjusted for different steroid regimen.

Supplementary Table 4 Characteristics of steroid maintenance and steroid withdrawal groups after propensity score matching

	Steroid maintenance (n = 65)	Steroid withdrawal (n = 65)	p value	SMD
Female	29 (44.6%)	30 (46.2%)	1	0.031
Characteristics at MG Onset				
Age of MG onset	48.7 ± 15.1	49.4 ± 14.2	0.793	0.046
MGFA classification			0.713	
I	39 (60.0%)	44 (67.7%)		
II	16 (24.6%)	12 (18.5%)		
III	9 (13.8%)	7 (10.8%)		
IV	0	1 (1.5%)		
V	1 (1.5%)	1 (1.5%)		
MG-ADL at steroid initiation	6.0 (4.0, 7.0)	5.0 (4.0, 8.0)	0.846	0.009
Other Autoimmune Diseases	9 (13.8%)	6 (9.2%)	0.583	
Antibody			0.884	0.087
AChR	52 (80.0%)	52 (80.0%)		
MuSK	3 (4.6%)	2 (3.1%)		
Seronegative	10 (15.4%)	11 (16.9%)		
Thymoma	13 (20.0%)	14 (21.5%)	1	0.038
Thymectomy	17 (26.2%)	17 (26.2%)	1	<0.001
Steroids regimen			0.952	0.055

Regimen 1: High Start, Fast Taper	7 (10.8%)	6 (9.2%)		
Regimen 2: Moderate Start, Gradual Taper	34 (52.3%)	34 (52.3%)		
Regimen 3: Low Start, Slow Taper	24 (36.9%)	25 (38.5%)		
Time from steroid initiation to first taper to maintenance dose or withdrawal (month)	32.0 (22.0, 46.0)	34.0 (16.0, 53.0)	0.751	0.018

Supplementary Table 5 Side effects of steroids maintenance and steroids withdrawal groups after propensity score matching

	Steroids maintenance (n = 65)	Steroids withdrawal (n = 65)	p value
Hypertension	11 (16.9%)	5 (7.7%)	0.181
Diabetes	8 (12.3%)	11 (16.9%)	0.620
Insomnia requiring medication	9 (13.8%)	10 (15.4%)	1
Excess body weight			0.116
Overweight	11 (16.9%)	7 (10.8%)	
Obesity	10 (15.4%)	4 (6.2%)	
Infection	20 (30.8%)	4 (6.2%)	**<0.001**
Specific infectious diseases			0.222
Pulmonary infection	4 (6.2%)	1 (1.5%)	
Urinary tract infection	3 (4.6%)	0	
Skin infection	1 (1.5%)	1 (1.5%)	
Herpes zoster	1 (1.5%)	1 (1.5%)	
Gastrointestinal infection	0	0	
Tuberculosis	1 (1.5%)	0	
Central nervous system infection	0	0	
Reduced bone density			0.505
Osteopenia	20 (30.8%)	14 (21.5%)	
Osteoporosis	11 (16.9%)	15 (23.1%)	
Osteoporotic Fractures	5 (7.7%)	3 (4.6%)	

Femoral head necrosis	3 (4.6%)	2 (3.1%)	1
Cataract requiring surgery	4 (6.2%)	4 (6.2%)	1

CTCAE, Common Terminology Criteria for Adverse Events.
